# Factors Associated with Anemia among Adults and the Elderly Family Farmers

**DOI:** 10.3390/ijerph19127371

**Published:** 2022-06-16

**Authors:** Sílvia Oliveira Lopes, Sarah Aparecida Vieira Ribeiro, Dayane de Castro Morais, Elizangela da Silva Miguel, Laís Silveira Gusmão, Sylvia do Carmo Castro Franceschini, Silvia Eloiza Priore

**Affiliations:** 1 Department of Nutrition and Health, Federal University of Viçosa, Viçosa 36570-900, MG, Brazil; sarahvieiraribeiro@gmail.com (S.A.V.R.); dayanecm@yahoo.com.br (D.d.C.M.); elizangela.silva1111@gmail.com (E.d.S.M.); sylvia@ufv.br (S.d.C.C.F.); sepriore@ufv.br (S.E.P.); 2 Postgraduate Program in Memory: Language and Society, State University of Southwest Bahia, Vitória da Conquista 45030-900, BA, Brazil; laigusmao@yahoo.com.br

**Keywords:** iron deficiency, hemoglobin, anemia, nutritional status, rural environment

## Abstract

The majority of studies on anemia are focused on children and women of reproductive age. Although the disease is a widespread public health problem, studies that include the rural population are scarce. This study determined the prevalence of anemia and associated factors in adults and the elderly living in the rural area of a municipality in Minas Gerais. Twelve rural communities were included. During home visits, hemoglobin levels were measured using a hemoglobinometer to check for the presence or absence of anemia. Additionally, anthropometric data and food insecurity data based on the Brazilian Food Insecurity Scale (EBIA) were collected. A questionnaire about socioeconomic, demographic, and housing conditions was applied. Analyses were performed using the Stata software version 13.0. Spearman correlation and regression analysis logistics were performed (*p* < 0.05) on 124 families (*n* = 297 farmers). The prevalence of anemia was 41.1%, being higher among women (55.7%). Additionally, 40.1% of the farmers were food insecure; 52.7% and 80.5% presented excess weight and cardiovascular risk, respectively. Poverty was a reality for 39.7% of individuals. A positive correlation between hemoglobin levels and per capita income was found as well as a negative correlation with EBIA scores and cardiovascular risk. Multivariate analysis showed that individuals experiencing food insecurity, the elderly, and those who do not own a property, were more likely to be anemic. Farmers with per capita income above 1/2 minimum wage were less likely to have anemia. The prevalence of anemia in the group studied was higher than previous studies. The disease is associated with factors that also predispose to food insecurity. The improvement of the determinants of insecurity can contribute to the fight against anemia.

## 1. Introduction

Anemia is defined by the World Health Organization (WHO) as the “condition in which the hemoglobin concentration in the blood is lower than normal as a result of the deficiency of one or more essential nutrients”, which may be iron, zinc, vitamin A, D, B6, B9, B12, and protein [1]. The groups mostly affected are children, pregnant, breastfeeding, women of reproductive age, and the elderly [2]. 

The first signs of change in the nutritional status of iron can be observed through erythrocyte parameters, such as ferritin and subsequent changes in hemoglobin. Thus, the assessment of these parameters is necessary for the investigation of possible factors that culminate in decreased iron levels, especially because anemia is characterized by disorders during hemoglobinization, which may be caused by iron deficiency [3].

The monitoring of micronutrient deficiencies, including iron, has been highlighted in health strategies worldwide. The first assessments of iron deficiency were carried out in 1993 and served as a basis for discussions on preventive actions against the deficiency [1]. Iron deficiency is not always accompanied by anemia; however, the main cause of anemia is iron deficiency [4]. 

According to Kassebaum et al., the global prevalence of anemia is 27%, in other words, 1.93 billion people are affected by the disease, with developing countries accounting for 89% [4]. Anemia is widely distributed in the population and is considered a risk factor for morbidity and mortality mainly among children of preschool age, women of reproductive age, and the elderly [5,6,7]. Due to this high prevalence, the World Health Assembly has set a target of 50% reduction in the incidence of anemia among women of reproductive age by 2025 [6]. However, reduced investments in public policies aimed at combating (directly or indirectly) micronutrient deficiencies/hidden hunger coupled with the health crisis caused by the COVID-19 pandemic can make attaining this goal even more difficult [8].

In Brazil, the prevalence of anemia demonstrates the importance of evaluation and promotion of health interventions that combat the disease. According to data from studies such as the 2006 National Survey of Demographics and Health of Children and Women, anemia prevalence was 29.4% in women [5]. Additionally, the study of Machado et al. showed a prevalence of 9.9% in women [9], as well as a prevalence of 8.8% [10], 12.5% [11], and 38% [12], respectively, in Porto Alegre, Campina Grande, and Salvador.

Anemia is responsible for 9% of the total burden of disability worldwide, leading to reduction in work productivity, learning capacity, and significant loss of cognitive ability, with implications on health, economic, and social development [2,13].

Some factors related to increased prevalence of anemia include low socioeconomic status, low level of education, and being underweight during pregnancy [14,15]. These factors often trigger food and nutritional insecurity, which cuts across questions pertaining to the access, availability, utilization, and stability of basic food supply and that of goods and services [5,6].

It is worth noting that food insecurity in Brazil affects 36.7% of the population, being higher in rural areas (46.4%) compared to urban areas (35.1%) [16]. This disparity is attributed to the historical contexts of exclusion faced by the rural population, difficulties in the organization of public actions, and the lack of basic sanitation in rural areas, fostering vulnerability of this population [17].

Also related to food insecurity in the countryside are the lower consumption of vegetables, meat, and eggs, characterized by a monotonous diet. The nutritional guidelines for the population recommend eating a variety of foods to provide different types of nutrients. Monotony in food can lead to low levels of micronutrients and protein, which can lead to inadequate nutritional status such as hidden hunger; thus, contributing to deficiencies such as anemia [17,18].

Besides the conditions of anemia and the presence of food and nutritional insecurities, most of the studies that address this theme are conducted among children and women of reproductive age. Given that anemia is a widespread public health problem, studies that focus on the adult and rural population are scarce [9]. Accordingly, studies with this target population can contribute to knowledge needed to structure public actions, especially those related to the rural population. Thus, this study determined the prevalence of anemia and associated factors in adults and elderly residents of the rural area of a municipality in Zona da Mata, Minas Gerais of Brazil.

## 2. Materials and Methods

### 2.1. Study Design and Population

This study is a cross-sectional study which forms part of the baseline of a project conducted with rural families “Food and nutrition education in the context of production for self-consumption in the situation of food and nutrition (in) security among households in the rural area of Viçosa-Minas Gerais”. The age group of this study included adults (≥ >/20–59 years) and not (>/20 and >/59 years) living in rural areas. Pregnant women were excluded.

Sample estimation was performed with the OpenEpi^®^ version 3.01 program, using the following equation: *n* = [EDFF × Np(1 − p)]/[(d2/Z21 − α/2 × (N − 1) + p × (1 − p)]. For the size of the population (*n*), the total number of adults and the elderly living in the rural area of the studied city (*n* = 4915) was considered; 9.9% prevalence (p) of anemia in adults and the elderly [9], 5% permissible error (d); 95% confidence level; 1.96 standard score (Z) and study design effect (EDFF) of 2.0 for random samples from the rural area [19], resulting in 246 individuals. After considering an additional 20% for dropout, incomplete data and control of confounding factors, the final sample size was 297 individuals. Subsequently, invitations were sent to family farmers. This study was carried out in 2019.

### 2.2. Ethical Standards Disclosure

This study was conducted according to the guidelines laid down in the Declaration of Helsinki and all procedures involving human subjects were approved by the Human Research Ethics Committee of Federal University Viçosa (1.052.838/2015). All procedures were adopted in accordance with the norms covering research with human beings [20]. The written consent was obtained from all participants. For those who could not read and write, the consent form was read by the researcher and the volunteer’s signature was digitally collected.

### 2.3. Assessment of Anemia

To detect anemia, hemoglobin level was measured at the participant’s home using a portable hemoglobinometer, brand HemoCue Hb 201^®^ (Hemocue AB—Ängelholm, Sweden). With the aid of a disposable blood lancet, a drop of blood was obtained from the right ring finger. For cases where drawing blood from this finger was impossible, the pinky finger was chosen. The first two drops of blood were discarded, and the third was evaluated. The blood sample was introduced into a microcuvette cavity and subsequently read on the hemoglobinometer. For the diagnosis of anemia, the cutoff points recommended by the World Health Organization for adults and the elderly were used: non-pregnant women: <12 g/dL and men < 13 g/dL [21].

### 2.4. Assessment of Food Insecurity

For the assessment of food insecurity, the Brazilian Food Insecurity Scale (EBIA) was used. EBIA is a scale consisting of 14 questions related to the experience of food insecurity, with yes or no answers. The first eight questions are asked in all households. The other questions are asked only in households with members less than 18 years of age. Each “yes” answer is equivalent to one point. At the end of the survey, the sum of the points was used as a basis for the classification of food security and insecurity [22].

### 2.5. Anthropometric Assessment

Weight, height, and waist circumference were measured considering the standards recommended by the Ministry of Health [23]. For weight measurement, a portable digital electronic scale (Marte^®^, Santa Rita do Sapucaí, Minas Gerais, Brazil) was used, with a capacity and precision of 200 kg and 100 g, respectively. Height was measured with a portable compact stadiometer (2 m) (AlturExata^®^, Belo Horizonte, Minas Gerais, Brazil). Waist circumference was measured with a 200 cm inelastic tape at the navel during normal expiration. The measurement was conducted in triplicate and the average value was calculated.

Body Mass Index (BMI) was calculated, using the cutoff points recommended for adults [24] and the elderly [25] as a diagnostic criterion. Waist to height ratio (WHR) was calculated, adopting a cutoff value ≥ 0.5 for cardiovascular risk [26].

### 2.6. Socioeconomic, Demographic, and Housing Conditions

In order to characterize the socioeconomic, demographic, and housing conditions of the participants, a semi-structured questionnaire was applied by a trained interviewer at the individuals’ homes. Information regarding sex, age, number of residents, education, income, and housing characteristics was collected [27].

To classify poverty, total income declared was divided by the number of residents. Poverty was defined as having a per capita income below 1/2 minimum wage/month [18].

### 2.7. Statistical Analysis

The data were entered in Microsoft Office Excel^®^ 2010 using the double entry method and followed by validation of conflicting responses. For the analysis, the Stata^®^ software version 13.0 was used. For the characterization of categorical variables, the distribution of absolute and relative frequencies was used. The distribution of variables was determined by the Shapiro–Wilk test.

The Spearman correlation test was applied to assess the correlation between hemoglobin levels and other numerical variables.

Binary logistic regression was performed to assess the association between predictor variables and the presence of anemia. The variables that presented *p* < 0.20 in the bivariate analyses were included in the multiple models, to estimate the odds ratios and 95% confidence intervals. In the final models, variables that were associated with the dependent variable (*p* < 0.05) were considered.

## 3. Results

The sample consisted of 297 people, aged 20–93 years and average age of 51.4 (±17.2) years. The majority (52.5%, *n* = 156) of the participants were female. The prevalence of anemia was 41.1% (*n* = 122), of which 55.7% (*n* = 68) were women. Of the total adult population assessed (*n* = 198), 36.0% were anemic and among the elderly, 50.5% (*n* = 99) were anemic.

According to the assessment of food insecurity based on EBIA, 40.1% (*n* = 119) were food insecure; 52.7% (*n* = 156) of the individuals evaluated were overweight and 80.5% (*n* = 235) presented cardiovascular risk based on WHR. In total, 39.7% (*n* = 118) of the individuals were living in poverty (Table 1).

**Figure 1:**
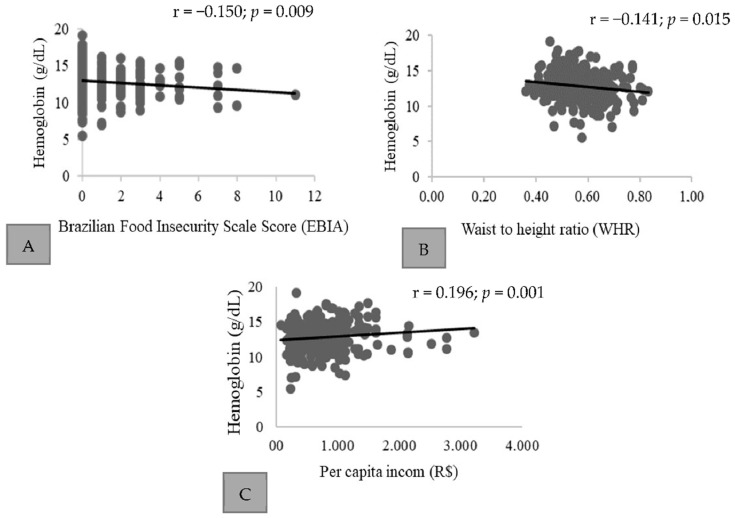
Correlation between hemoglobin values (g/dL), EBIA, WHR, and per capita income (BRL) of adults and elderly in rural households in a municipality of Brazil. Correlation was found between hemoglobin values, EBIA scores (**A**), and WHR values (**B**), while per capita income presented a positive correlation (**C**).

A negative correlation was found between hemoglobin values, EBIA scores (Figure 1A), and WHR values (Figure 1B), while per capita income presented a positive correlation (Figure 1C).

All the explanatory variables presented in Table 2 were included in the final model, which presented *p* < 0.20 in the simple analysis. Table 3 shows the final model of the multiple logistic regression analysis, where individuals more likely to be anemic were those in a situation of food insecurity, the elderly, and individuals who do not own a home. People with income greater than 1/2 minimum wage per capita were less likely to have anemia.

## 4. Discussion

In this study, the prevalence of anemia among individuals older than or equal to 20 years, corresponding to adults and the elderly was 41.1%. This value is higher than those found in the national data (9.9%) for the same population group [9]. A prevalence greater than 40% characterizes a serious public health problem [24], requiring rapid intervention actions.

The elderly are at risk of developing anemia because aging involves physiological and functional changes that can lead to the disease [28,29]. Among the elderly, anemia is associated with an increased risk of death, impaired muscle function, and dementia [30]. However, aging should not be considered a sole triggering factor since other factors may interfere with the progression of anemia, such as diet [14,31,32].

In relation to adults, studies and policies to control anemia are especially geared towards women of reproductive age as they are considered a risk group with a prevalence of 29.4% according to national studies, and a global prevalence of 29% in non-pregnant women [5,33]. It should be noted that the progression of anemia affects individual productivity, influencing economic status [33]. Therefore, in view of the findings, it is necessary to organize health actions focused on this age group.

The impairment of adults and the elderly by anemia runs through productivity issues as the disease affects the functional capacity of tissues and compromises the transport of oxygen to the red cells, which alters energy efficiency. This is because iron transports and uses oxygen in the production of energy, leading to a decrease in individual productive capacity [33].

An important factor that should be highlighted is the relationship between the presence of anemia and food insecurity. In the literature, this relationship is elucidated with risk groups, such as: children of preschool age, women of reproductive age, and the elderly [7,15]. Adults are rarely addressed, where the presence of the disease is in many cases associated with food insecurity, due to the lack of access to food in sufficient quantity and quality, compromising the availability of micronutrients, which can trigger hidden hunger [18].

The prevalence of food insecurity is also associated with lower income, low level of education, number of residents in the household, absence of treated water, type of housing, and place of residence. Families living in rural areas are more likely to be food insecure, which corroborates data from the National Household Sample Survey (2014) [16], which reports a higher prevalence of food insecurity in the rural population compared to the urban population [15,17].

The lack of access to goods and services as found in this work as “not owning home”, increases the risk of anemia, since priority may be given to essential necessities, such as housing, to the detriment of others such as quality of food, characterizing food insecurity. As also noted, higher income is a protective factor against anemia, which further strengthens the understanding of the social factors leading to food insecurity [22].

A study carried out with a group of children showed a greater chance of anemia among families who do not own a house or those renting and presenting a high number of residents, being that low purchasing power would also be related to low availability of food and lower food variety, resulting in insufficient consumption and low bioavailability of nutrients, including iron [34].

The rural population has other risk factors such as lower consumption as fruits and vegetables compared to urban residents. Contrary to the idea that the rural population would have a higher consumption because it is a place where food production takes place. This lower consumption is due to cultural and financial factors. Thus, it is important to encourage agricultural practices that allow the rural population to be less susceptible to market variations, for example, production for self-consumption, encouraging family farming and solidarity economy, since access to food requires purchasing power [35,36,37].

Food and nutrition education is one of the strategies suggested to increase the population’s knowledge about healthy eating, aiming at health promotion. Encouraging practices that favor greater bioavailability of iron in the diet is a way to prevent anemia, for example, associating the consumption of a food source, meat, and an absorption facilitator such as vitamins C and A. In addition, a lower consumption of absorption inhibitors of iron, phytates, polyphenols, calcium, and phosphates also contribute to the prevention of anemia. Monotonous diets, rich in cereals, roots, and tubers, increase the chances of micronutrient deficiencies. This eating pattern, characterized by monotony, is a factor of concern that is already a reality of individuals experiencing food insecurity [38,39].

Thus, actions are required to encourage diets with nutritional and cultural quality, allowing the construction of healthy environments and encouraging production for self-consumption as a promoting factor of food and nutritional security. The rural environment is seen as a health-promoting environment; thus, these initiatives can act to combat nutritional deficiencies, such as anemia [35,37].

In many cases, the population in the countryside is marginalized in relation to coverage and access to health services, as well as actions that promote food and nutritional security which run through economic and social actions that highlight the exclusion of the countryside. A study conducted on male and female farmers showed a 24.8% prevalence of anemia [35] with the help of a hemoglobinometer, an instrument recommended by the World Health Organization for diagnosis and development of intervention strategies [1]; thus, contributing to early anemia diagnosis. 

The findings of the present study justify the importance of promoting public health actions, especially with the rural population, since few studies focus on anemia among adults and elderly family farmers, and this study addresses a group not previously studied.

A limitation of this study was the lack of investigation of other biochemical parameters that help to identify the causes of anemia in this population group. However, all farmers identified with anemia were referred and monitored for four months by a multidisciplinary team for the treatment of the disease, with no other types of anemia being identified, only iron deficiency.

## 5. Conclusions

The prevalence of anemia in the studied group was higher than previous studies. The disease is associated with factors that also predispose to food insecurity. Therefore, public actions and policies to combat anemia are needed, especially in the rural population, where social inequality and difficulties in accessing health services are still a relevant factor for the aggravation of the disease. Improving the determinants of food insecurity can contribute to the control of anemia.

## Figures and Tables

**Table 1:** Food security, nutritional status, socioeconomic, demographic, and housing conditions of adults and the elderly in a rural municipality of Brazil.

Variables	Total (*n* = 297)	
	*n*	%
**Food Security Status**		
Secure	178	59.9
Insecure	119	40.1
Nutritional status *		
Normal	140	47.3
Excess weight ^1^	156	52.7
Waist/height ratio *		
Unaltered	57	19.5
Altered	235	80.5
**Age group**		
Adult	198	66.7
Elderly	99	33.3
**Sex**		
Female	156	52.5
Male	141	47.5
**Income ^2^**		
≤1/2 minimum wage	118	39.7
>1/2 minimum wage	179	60.3
Level of education (years) *		
≤4	178	60.1
>4	118	39.9
**Home owner**		
Yes	257	86.5
No	40	13.5
Number of residents *		
≤3	155	52.4
>3	141	47.6
**Number of rooms**		
≤4	105	35.4
>4	192	64.6
Access to sanitation service *		
Yes	25	8.4
No	271	91.6
**Water supply**		
Well	244	82.2
Spring	53	17.8
**Water treatment (filtered/boiled)**		
Yes	232	78.1
No	65	21.9
Waste disposal *		
Public waste collection	14	4.8
Burning/“open-air garbage dump”/Burying	279	95.2
**Gas stove**		
Yes	275	92.6
No	22	7.4

^1^ Excess weight = overweight + obese; ^2^ established minimum wage BRL 1045.00; * different answers *n* = 297.

**Table 2:** Prevalence of anemia in rural households in a municipality of Brazil, according to the assessment of food security, nutritional status, socioeconomic, demographic, and housing conditions.

Variables	Anemic (*n* = 122)		Not Anemic (*n* = 175)		OR CI 95%	*p* Value ^ǂ^
	*n*	%	*n*	%		
**Food Insecurity Status**						
Secure	61	50.0	117	66.9	2.017 (1.255–3.242)	0.004
Insecure	61	50.0	58	33.1		
Nutritional status *						
Normal	59	48.8	81	46.3	0.905 (0.569–1.440)	0.675
Excess weight ^1^	62	51.2	94	53.7		
Waist/height ratio *						
Not altered	25	20.7	32	18.3	0.884 (0.493–1.586)	0.679
Altered	96	79.3	139	79.4		
**Age group**						
Adult	72	59.0	126	72.0	1.786 (1.095–2.912)	0.020
Elderly ^2^	50	41.0	49	28.0		
**Sex**						
Female	68	55.7	88	50.3	1.245 (0.783–1.980)	0.355
Male	54	44.3	87	49.7		
**Income ^3^**						
≤1/2 minimum wage	60	49.2	58	33.1	0.512 (0.319–0.823)	0.006
>1/2 minimum wage	62	50.8	117	66.9		
Level of education (years) *						
≤4	84	68.9	94	54.0	0.532 (0.327–0.864)	0.011
>4	38	31.1	80	46.0		
**Home owner**						
Yes	98	80.3	159	90.9	2.434 (1.232–4.808)	0.010
No	24	19.7	16	9.1		
Number of residents *						
≤3	58	47.5	97	55.7	1.390 (0.874–2.212)	0.165
>3	64	52.5	77	44.3		
**Number of rooms**						
≤4	42	34.4	63	36.0	1.071 (0.659–1.739)	0.780
>4	80	65.6	112	64.0		
Access to sanitation service *						
Yes	13	10.7	13	7.4	0.673 (0.300–1.507)	0.335
No	109	89.3	162	92.6		
**Water supply**						
Well	93	76.2	151	86.3	1.962 (1.077–3.573)	0.028
Spring	29	23.8	24	13.7		
**Water treatment (filtered/boiled)**						
Yes	95	77.9	137	78.3	1.025 (0.586–1.791)	0.932
No	27	22.1	38	21.7		
Waste disposal *						
Public waste collection	5	4.1	9	5.3	1.3 (0.425–3.979)	0.646
Burning/“open-air garbage dump”/Burying	117	95.9	162	94.7		
**Gas stove**						
Yes	113	92.6	162	92.6	0.993 (0.410–2.400)	0.987
No	9	7.4	13	7.4		

^1^ Excess weight = overweight + obese; ^2^ reference of elderly (adults ≥ 20 years and <59 years; elderly ≥ 60 years); ^3^ established minimum wage BRL 1045.00; * different answers *n* = 297. OR = odds ratio; CI: confidence interval; ^ǂ^ binary logistic regression.

**Table 3:** Final logistic regression model of the association between anemia, food insecurity classified by the Brazilian Food Insecurity Scale (EBIA), being elderly, income and housing characteristics of rural households in a municipality of Brazil.

Variables	OR	CI 95%	*p* Value
Food insecurity	2.311	1.379–3.869	0.001
Elderly	2.861	1.642–4.983	<0.001
Not owning a home	2.651	1.237–5.681	0.012
Income ≤ 1/2 minimum wage	0.531	0.309–0.910	0.021

OR = odds ratio; CI: confidence interval.
